# Sequential Injection Analysis of Cholesterol Using an Oxidation–Reduction Electrode Detector

**DOI:** 10.3390/s25185863

**Published:** 2025-09-19

**Authors:** Takato Imanaka, Takashi Masadome

**Affiliations:** Department of Applied Chemistry, Shibaura Institute of Technology, Koto-ku, Tokyo 135-8548, Japan

**Keywords:** sequential injection analysis, potentiometry, oxidation–reduction electrode, cholesterol

## Abstract

A new automated method for the determination of cholesterol in serum was developed by combining sequential injection analysis (SIA) with potentiometric detection using a gold oxidation–reduction potential (ORP) electrode because serum cholesterol is an important indicator of abnormal lipid metabolism, arteriosclerosis, and hypertension in clinical diagnosis. The method is based on enzymatic hydrolysis of cholesterol esters by cholesterol esterase (CE) to yield free cholesterol, followed by oxidation with cholesterol oxidase (COD) to produce hydrogen peroxide. In the presence of horseradish peroxidase (HRP) and potassium ferrocyanide (K_4_[Fe(CN)_6_]), hydrogen peroxide oxidizes ferrocyanide to ferricyanide (K_3_[Fe(CN)_6_]), and the concentration ratio of ferri-/ferrocyanide is determined potentiometrically. Experimental conditions were optimized as follows: 5.0 mM K_4_[Fe(CN)_6_], 2 min reaction time, 0.5 units/mL HRP, 0.75 units/mL COD for free cholesterol, 1.5 units/mL COD and 10.0 units/mL CE for total cholesterol, and 5.0% (*w*/*v*) Triton X-100 with 5.0% (*v*/*v*) isopropanol as solubilizing agents. Under these conditions, the calibration curve for total cholesterol exhibited a Nernstian slope of 47.6 mV/decade over the range of 1.0 × 10^−5^–1.0 × 10^−3^ M, with no significant interference from common serum constituents. This method offers low reagent consumption, high automation, and simple operation, making it promising for clinical cholesterol analysis.

## 1. Introduction

Serum cholesterol is an important biomarker for diagnosing dyslipidemia, atherosclerosis, and hypertension [1]. Therefore, there is high demand for analytical methods that can quickly, easily, and reliably measure cholesterol. Useful techniques, such as gas chromatography, high-performance liquid chromatography, electrospray ionization tandem mass spectrometry, and matrix-assisted laser desorption/ionization mass spectrometry imaging, can achieve highly accurate and sensitive quantification [2,3]. However, these methods require large, expensive equipment and time-consuming sample preparation, which limits their routine use in clinical laboratories.

A promising approach to overcoming these limitations is the use of electrochemical detection systems based on amperometric biosensors that utilize enzymatic reactions [4,5,6,7]. These systems offer advantages in terms of miniaturization and simplicity with potential clinical applications.

Flow injection analysis (FIA) is an automated analytical technique that enables rapid and reproducible measurements by injecting samples into a continuous carrier flow. The samples are mixed with reagents and reaction products are detected downstream. Combined with flow-based technologies like FIA, these amperometric biosensors enable rapid, reproducible cholesterol measurements [8,9,10,11,12,13,14,15]. However, FIA requires a continuous flow of both the carrier and reagent solutions, which increases reagent consumption and waste generation.

To overcome this limitation, SIA was developed [16,17,18,19,20,21]. SIA is an automated analytical method that uses a syringe pump (SP) and a computer-controlled, multi-port selector valve (SV) to introduce sample and reagent solutions sequentially into a holding coil (HC). This induces mixing and chemical reactions via bidirectional flow. The reaction products are then sent to a detector for measurement. SIA is very useful for multi-step analyses involving enzymes or antibodies, enabling the automation of complex analytical protocols while minimizing reagent consumption and waste. SIA has already been applied to cholesterol measurement with absorbance detection [22].

Along with advances in flow analysis technology, the selection of detection principles is also critically important. Potentiometric analysis, which is based on ion-selective electrode (ISE), is widely used in clinical laboratories to measure electrolytes such as Na^+^, K^+^, and Cl^−^. Importantly, potentiometric detection can be adapted to measure biomolecules, such as cholesterol, by monitoring the redox reactions of enzymatically generated species (e.g., hydrogen peroxide).

We have previously reported on SIA methods for measuring lactate and cholinesterase using potentiometric detection based on ISE [23,24]. On the other hand, the FIA method employing a chemically stable ORP electrode, such as tungsten, has been applied for cholesterol analysis [25]. However, this FIA system lacked sufficient automation of the enzymatic reaction steps, which limited the full automation of cholesterol quantification.

This study aims to develop a fully automated cholesterol analysis method that combines the benefits of SIA with those of potentiometric detection. Specifically, we propose an SIA system that uses a gold electrode as the ORP detector for measuring cholesterol. We selected the gold electrode due to its high stability, reproducibility, and distinct redox characteristics, which are expected to enhance the robustness and clinical applicability of cholesterol analysis.

## 2. Experimental

### 2.1. Reagents

The following were obtained from Fujifilm Wako Pure Chemical Corporation, Osaka, Japan: HRP (horseradish peroxidase, 100 units/mg), precision control serum I (BR) and precision control serum lipid control serum set, CE for biochemistry and COD. Cholesterol (specification content: 99.5%+) was obtained from Nagara Science Co., Ltd., Gifu, Japan. All other reagent-grade chemicals were obtained from Sigma-Aldrich Japan, Tokyo, Japan or Fujifilm Wako Pure Chemical Corporation and were used without further purification. Milli-Q water was used in all experiments.

### 2.2. SIA System

**Figure 1:**
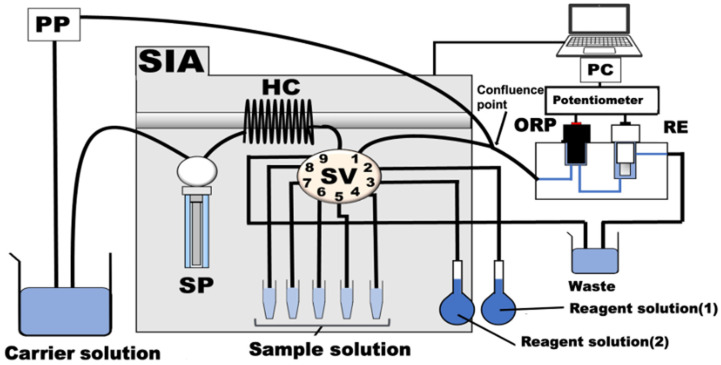
Schematic diagram of the SIA system for the determination of cholesterol. SP: syringe pump, SV: nine-port selection valve, HC: holding coil: i.d. 0.5 mm × 480 cm, PP: peristaltic pump. ORP: gold electrode, RE: reference electrode. All instruments were connected by Teflon tubing with an inner diameter of 1.0 mm. Carrier solution: 10 mM phosphate (Na_2_HPO_4_/NaH_2_PO_4_) buffer solution with containing 5.0 mM K_4_[Fe(CN)_6_] (pH 7.0), reagent solution (1): 0.5 unit/mL HRP + 5.0 mM K_4_[Fe(CN)_6_], reagent solution (2): 1.5 units/mL COD + 10.0 units/mL CE.

The serum cholesterol SIA measurement system consists of an SP, an SV, an HC, a potentiometric detector with gold and reference electrodes, a potentiometer, a peristaltic pump (PP), and a PC, as shown in Figure 1. Table 1 shows protocols of the experimental procedures for the determination of total cholesterol. First, the carrier solution (phosphate-buffer solution, pH 7.0) is aspirated from the SP and sent to the detector via port 1 of the SV to clean the interior of the 1 mm tubes at a flow rate of 29.0 µL/s. Next, the serum sample of interest (125 µL) is aspirated from ports 6 to 8 of the SV. The insides of the tubes are cleaned, and the sample is disposed of as effluent through port 9 with the carrier solution (500 µL). Then, the following solutions were aspirated into the HC: reagent solution (1) (125 µL), consisting of cholesterol esterase (CE) and cholesterol oxidase (COD) from port 2; reagent solution (2) (125 µL), consisting of horseradish peroxidase (HRP) and potassium ferricyanide (K_4_[Fe(CN)_6_]) from port 3; and the target sample (the serum solution, 125 µL) from ports 4 to 8. To react the reagents and sample solution, the mixed solution in the HC was repeatedly sent from port 1 of the SV to the detector and back. Finally, the flow was stopped between port 1 and the confluence point. The obtained reaction product (K_3_[Fe(CN)_6_]) was then aspirated into the HC and pumped to the detector with the carrier solution. The potential difference between the ORP electrode and the RE in a potentiometric detector (FLC11, TOA-DKK) was measured as a peak signal with a potentiometer (EPU353 pH and ISE USB IsoPod™). To stabilize the potential of the potentiometric detector, the carrier solution was continuously pumped from the PP at a flow rate of 2.95 µL/s to the detector. A PC controls and automates all of these operations.

## 3. Results and Discussion

**Scheme 1:**
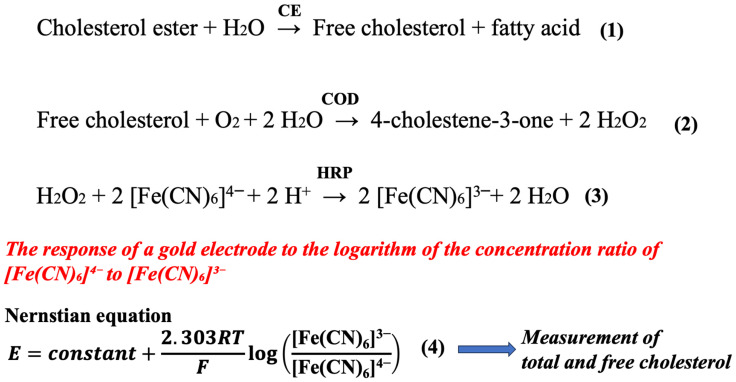
As can be seen from the above equations, the cholesterol measurement reaction consists of reactions (1)–(4). These reactions involve hydrogen peroxide, which is generated by reaction (2), as well as HRP and [Fe(CN)_6_]^4−^.

The principle of cholesterol measurement is shown in Scheme 1. Total cholesterol consists of cholesterol esters and free cholesterol. Cholesterol esters are converted to free cholesterol in the presence of CE. Free cholesterol is then oxidized by COD to produce hydrogen peroxide. In the presence of HRP, the hydrogen peroxide is reduced by [Fe(CN)_6_]^4−^ to form [Fe(CN)_6_]^3−^. The reactant product from cholesterol or its ester is stoichiometrically converted to [Fe(CN)_6_]^3−^. The gold electrode detector responds to the logarithm of the concentration ratio of [Fe(CN)_6_]^4−^ to [Fe(CN)_6_]^3−^, resulting in the measurement of total and free cholesterol.

We first examined the response behavior of the gold electrode to the concentration ratio of [Fe(CN)_6_]^4−^ and [Fe(CN)_6_]^3−^ using the SIA method with a gold electrode detector. For this study, we connected 0.1, 0.2, 0.6, and 1.0 mM solutions of K_3_[Fe(CN)_6_] containing a 5.0 mM solution of K_4_[Fe(CN)_6_] as the samples to ports 2–5 of the SV in Figure 1. These mixed solutions were then fed to the potentiometric detector. The results showed a good Nernstian potential response of 59.2 mV/decade between the peak height obtained by the gold electrode detector and the logarithm of [Fe(CN)_6_]^3−^ in the concentration range of 0.1, 0.2, 0.6, and 1.0 mM. These results confirm the applicability of the SIA method with a gold electrode detector for measuring the [Fe(CN)_6_]^4−^/[Fe(CN)_6_]^3−^ concentration ratio.

Because the sensitivity of cholesterol detection depends on the sensitivity of hydrogen peroxide produced by reactions (1) and (2), we examined how [Fe(CN)_6_]^4−^ concentration and reaction time affect hydrogen peroxide detection sensitivity. The concentrations of HRP and hydrogen peroxide were 0.1 units/mL and 0.50 mM, respectively. Additionally, we varied the K_4_[Fe(CN)_6_] concentration from 0.1 to 5.0 mM and the reaction time from 0 to 5 min. In this case, K_4_[Fe(CN)_6_], hydrogen peroxide and HRP solutions were connected to ports 3–5 of the SV in Figure 1, respectively. The peak height of hydrogen peroxide at a K_4_[Fe(CN)_6_] concentration of 4.0–5.0 mM with a reaction time of two minutes was found to be equivalent to that at a K_4_[Fe(CN)_6_] concentration of 1.0–2.0 mM with a reaction time of five minutes. Considering the balance between measurement time and sensitivity, subsequent examinations used a two-minute reaction time and a 5.0 mM concentration of K_4_[Fe(CN)_6_]. The sensitivity of hydrogen peroxide detection from reaction (3) depends on the HRP concentration. Therefore, we examined how HRP concentration affects hydrogen peroxide measurement sensitivity. In this case, K_4_[Fe(CN)_6_], hydrogen peroxide and HRP solutions were also connected to ports 3–5 of the SV in Figure 1, respectively. Sensitivity to hydrogen peroxide increased linearly with a logarithmic increase in HRP concentration between 0.010 and 0.10 units/mL. However, when the HRP concentration was between 0.10 and 1.0 units/mL, sensitivity remained nearly constant. Based on these results, we used an HRP concentration of 0.5 units/mL for subsequent examinations.

**Figure 2:**
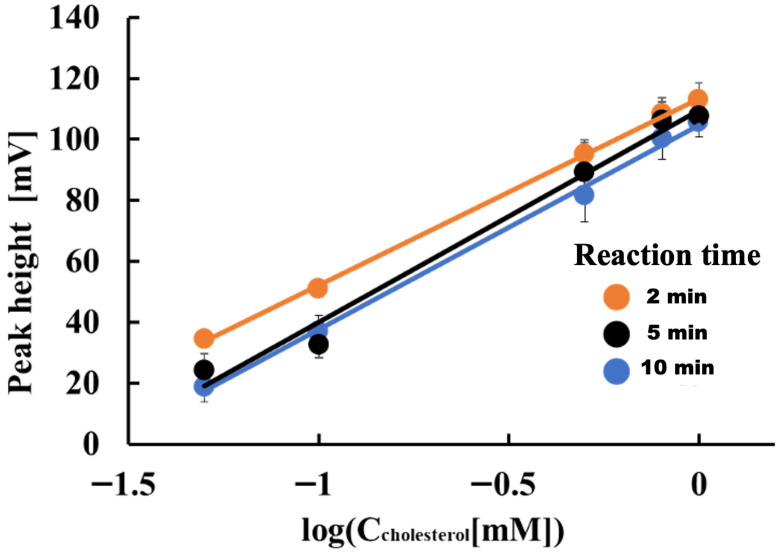
Effect of reaction time on determining free cholesterol.

Next, we examined how reaction time affects the determination of free cholesterol. The reagents used in this case were 5 mM K_4_[Fe(CN)_6_], 0.5 units/mL HRP, and 1.0 units/mL COD. Figure 2 shows the results. The electrode detector’s detection sensitivity for cholesterol in the 0.05 to 1.0 mM concentration range was 61.3, 69.6, and 67.1 mV/decade at reaction times of 2, 5, and 10 min, respectively. The lowest detection sensitivity was observed at a reaction time of two minutes. However, given the analysis throughput, the reaction time was set to two minutes for subsequent studies.

**Figure 3:**
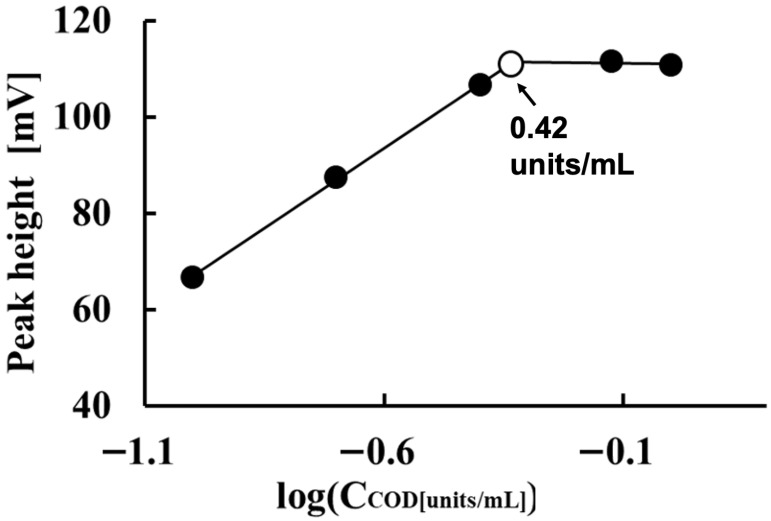
Effect of COD concentration on measuring 1.0 mM free cholesterol.

Figure 3 shows the effect of COD concentration on measuring 1.0 mM free cholesterol. In this case, 5 mM K_4_[Fe(CN)_6_] and 0.5 units/mL HRP were used as the respective reagents. As the COD concentration increased from 0.10 to 0.42 units/mL, the electrode detector’s sensitivity to free cholesterol increased proportionally to the logarithm of the cholesterol concentration. However, when the COD concentration exceeded 0.42 units/mL, the electrode detector’s sensitivity to 1.0 mM free cholesterol only increased by a few millivolts as the COD concentration increased from 0.42 to 0.75 and 1.0 units/mL. The electrode detector’s sensitivity to 1.0 mM free cholesterol was highest at a COD concentration of 0.75 units/mL. Based on these results, we set the COD concentration for quantifying free cholesterol at 0.75 units/mL.

**Figure 4:**
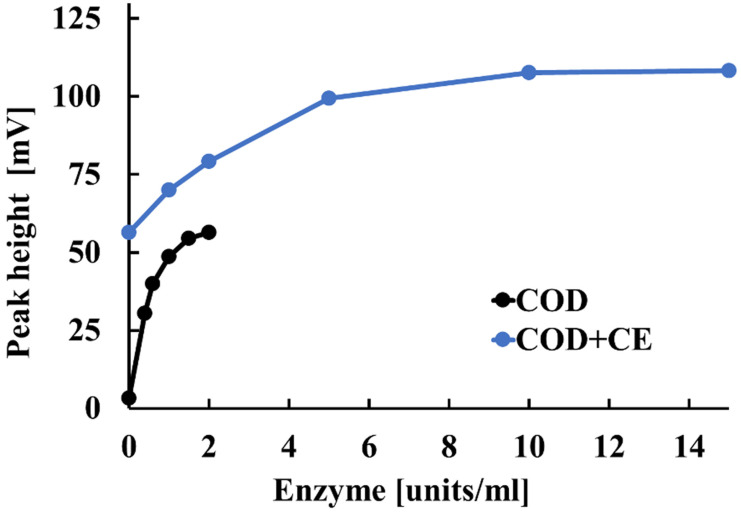
Effect of CE and COD concentrations on the electrode detector’s sensitivity to cholesterol esters and free cholesterol in serum.

Next, we examined how CE and COD concentrations affect the sensitivity of the electrode detector to cholesterol esters and free cholesterol in serum. Figure 4 illustrates these effects. For COD concentrations between 0 and 1.0 units/mL, the potentiometric detector’s peak height increased as the COD concentration increased. Above 1.5 units/mL, however, the peak height obtained from the potentiometric detector remained almost constant with increasing COD concentration. This is likely because the amount of free cholesterol reacting at COD concentrations above 1.5 units/mL reached saturation. Based on these results, the optimal COD concentration was determined to be 1.5 units/mL. A similar study was conducted for CE in the range of 0 to 15.0 units/mL. For the 0–10 units/mL range, the peak height obtained by the potentiometric detector increased with increasing CE concentration. For CE concentrations above 10 units/mL, the peak height obtained by the potentiometric detector remained almost constant, even with an increased CE concentration. This is thought to occur because the amount of CE reacting at concentrations above 10 units/mL reaches saturation. Based on these results, the optimal CE concentration was determined to be 10.0 units/mL.

The mechanistic interpretation of Figure 4 is as follows: If there is insufficient CE, the cholesterol esters are not completely hydrolyzed. This results in less free cholesterol and a reduced electrode response. Once sufficient amounts of CE are present, complete hydrolysis occurs, and adding more CE has no effect. Similarly, if COD is limited, hydrogen peroxide generation is reduced. However, once sufficient COD is supplied, the amount of hydrogen peroxide produced depends solely on the concentration of free cholesterol. Thus, when CE and COD are both present at optimal levels, the electrode response is governed exclusively by the total cholesterol concentration in the sample. The observed saturation in response corresponds to the point at which all available substrate has been converted.

**Figure 5:**
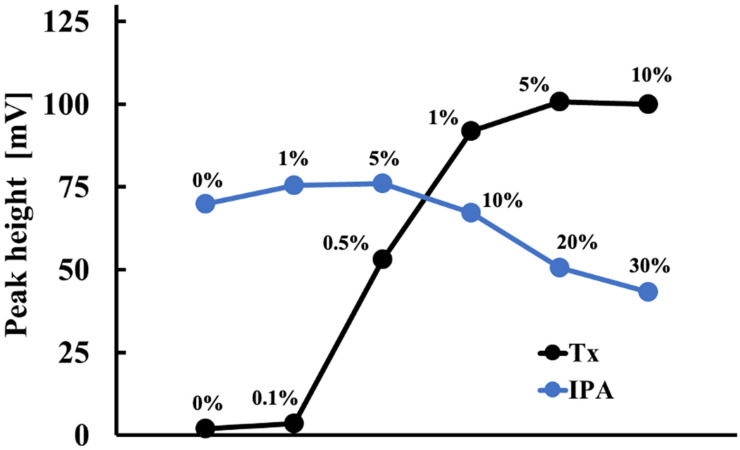
Effect of Tx and IPA concentrations on the measurement of 1 mM total cholesterol in serum.

Triton X-100 (Tx), a nonionic surfactant, and isopropanol (IPA) were found to effectively solubilize lipids in the cholesterol solution. Tx keeps the cholesterol and COD in solution, and IPA acts as an emulsifier. However, the optimal surfactant-to-cholesterol ratio must be determined by balancing the need to solubilize the cholesterol with minimizing interference with enzyme activity caused by the surfactant. Figure 5 illustrates the impact of Tx and IPA concentrations on the electrode detector’s sensitivity to 1 mM total cholesterol in serum. The electrode detector’s response sensitivity was maximum when the Tx and IPA concentrations were 5.0% (*w*/*v*) and 5.0% (*v*/*v*), respectively. Thus, a solution containing 5.0% (*w*/*v*) Tx and 5.0% (*v*/*v*) IPA was used to prepare the serum solution.

**Figure 6:**
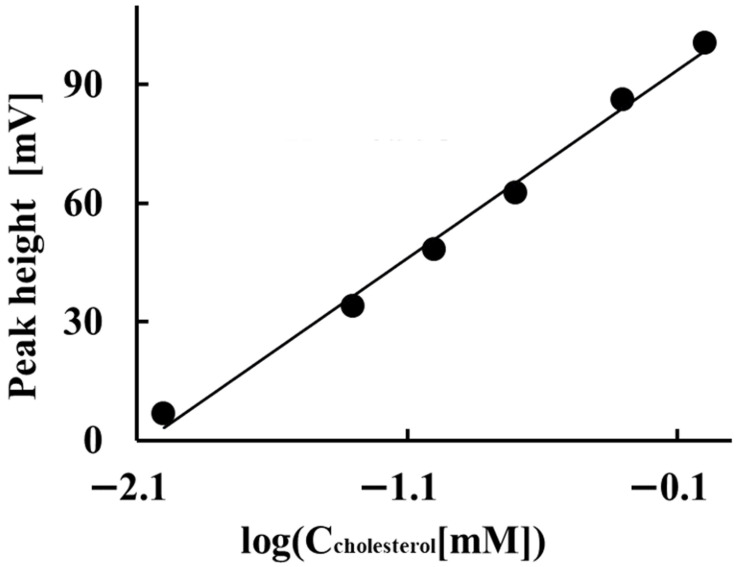
Potentiometric detector’s potential response to total cholesterol in control serum under these conditions.

Figure 6 shows the results of the investigation into the potentiometric detector’s potential response to total cholesterol in control serum under these conditions. The potentiometric detector exhibited a sub-Nernstian potential response of 47.6 mV/decade between the peak height and the logarithm of the total cholesterol concentration within the 1.0 × 10^−5^ to 1.0 × 10^−3^ M range. The limit of quantitation for the determination of total cholesterol in this method was 1.0 × 10^−5^ M. The limit of detection was approximately 9.0 × 10^−6^ M at a signal-to-noise ratio of 3.0. The relative standard deviations (CV) of the peak heights obtained by the ORP electrode for three same-day measurements of 0.05, 0.10, and 1.0 mM total cholesterol in serum were 2.3%, 2.9% and 0.16%, respectively. The CV of the peak height in three measurements of 1.0 mM total cholesterol on different days was 1.3%. These results indicate a high reproducibility of the electrode response obtained with this system: the drift of the baseline potential obtained by the ORP electrode was within a few mV per 3.3 h. The electrode’s response to cholesterol in the serum is lower than its Nernstian sensitivity. This may be due to the presence of other co-interfering substances in the serum, in addition to cholesterol. In 2020, the Japan Committee for Clinical Laboratory Standardization set the reference range for total cholesterol in serum at 3.67–6.41 mmol/L. Our method’s determination concentration range for total cholesterol in serum falls within this reference range.

Serum samples contain reducing agents, proteins, organic acids, and inorganic cations and anions. We investigated how these substances affect the quantification of 1.0 mM free cholesterol. Quantification was not affected by the presence of 0.2 mM bilirubin, 0.16 mM uric acid, 0.084 mM creatinine, 9.0 mM glucose, or 50 mg/mL albumin. These results demonstrate that substances present at serum concentrations do not interfere with cholesterol determination. However, ascorbic acid may interfere with cholesterol measurement. The interference can be eliminated through a two-step process. Initially, the cholesterol is reacted with ascorbic acid oxidase solution, which functions as the reagent solution. Subsequently, the resulting product is reacted with the subsequent reagent solution (1) and reagent solution (2) in Figure 1.

Table 2 compares the FIA and SIA methods for measuring total cholesterol using an electrochemical detector with amperometric and potentiometric techniques. The measurement concentration range and detection limit for total cholesterol with this method are equivalent to the values reported with other methods. However, this method achieves fully automated measurement of total cholesterol using the SIA method, unlike other methods. Amperometric biosensors are known to exhibit decreased detection sensitivity and a shorter lifespan with prolonged use due to enzyme detachment. Nevertheless, the gold electrode detector used in this study showed no performance degradation after three years of use. Based on this, this method is considered very useful for total cholesterol measurement.

## 4. Conclusions

This study developed an SIA method combined with potentiometric detection using a gold oxidation–reduction electrode to determine cholesterol levels in serum. This method offers the benefits of an automated SIA approach, including low reagent consumption and high throughput, while maintaining the simplicity and robustness of potentiometric measurement. Optimizing the reaction conditions, including the concentrations of the reagents (CE, COD, and HRP), the reaction time, and the solubilizing agents (Triton X-100 and isopropanol), produced a stable Nernstian response for both free and total cholesterol. The proposed method exhibited high sensitivity and selectivity, with minimal interference from common serum components. These results demonstrate that the SIA-potentiometry system is a rapid, reliable, and cost-effective approach for determining cholesterol, making it suitable for routine clinical and biochemical analyses. In the future, the OPR electrode can be used to measure the concentration of hydrogen peroxide generated by the reaction between the substrate and various oxidases (the oxidase system). It is expected that the proposed SIA-potentiometry system will be able to quantify not only cholesterol but also clinically important items such as lactic acid and uric acid simultaneously.

## Figures and Tables

**Table 1:** Protocols of the experimental procedures for the determination of total cholesterol.

Step	Position of SV	Volume (µL)	Flow Rate (µL/mL)	Event
1	1	937	29	Aspiration of carrier solution
2	4–8	125	29	Aspiration of serum sample solution
3	9	500	29	Disposal of the sample as effluent with the carrier solution
4	2	125	29	Aspiration of reagent 1 into HC
5	3	125	29	Aspiration of reagent 2 into HC
6	4–8	125	29	Aspiration of serum sample solution into HC
7	1	1312	29	Repetition of the propulsion of the solution in HC to the detector side, then returning to HC.
8	-	-	0	Stopping the flow between Port 1 and the PP convergence point
9	1	1312	29	Aspiration of the reaction product obtained (K_3_[Fe(CN)_6_]) into HC and propulsion to the detector along with the carrier solution and measurement

**Table 2:** FIA and SIA methods for the determination of total cholesterol using amperometric and potentiometric electrochemical detectors.

Flow System	Electrochemical Detector	Determination Range and LOD	References
FIA	Amperometry	5–100/100–400 µM	[8]
FIA	Amperometry	1.25–10 mM	[9]
FIA	Amperometry	10–40 µM	[10]
FIA	Amperometry	Up to 0.3 mM LOD: 95 µM	[11]
FIA	Amperometry	Up to 0.3 or 0.4 mM	[12]
FIA	Amperometry	0.6–5 mM LOD: 0.022 mM	[13]
FIA	Amperometry	Up to 31 mmol/L LOD: 2.6 µM	[14]
FIA	Amperometry	Up to 1.035 mM LOD: 10 µM	[15]
FIA	Potentiometry	0.05–3.0 mM LOD: 0.01 mM	[25]
SIA	Amperometry	5–5000/0.5–5000 µM	[26]
SIA	Potentiometry	0.01–1.0 mM LOD: 9 µM	This work

## Data Availability

The datasets used and analyzed during the current study are available from the corresponding author upon request.
